# Public Coverage of Postpartum Services for Immigrants

**DOI:** 10.1001/jamahealthforum.2025.0702

**Published:** 2025-04-25

**Authors:** Rachel Fabi, Katherine Narváez Mena, Arielle Desir, Margaret White, Laura Wherry, Maria Steenland

**Affiliations:** 1 Center for Bioethics and Humanities, SUNY Upstate Medical University, Syracuse, New York; 2 Norton College of Medicine, SUNY Upstate Medical University, Syracuse, New York; 3 School of Public Health, Brown University, Providence, Rhode Island; 4 Frank H. Netter MD School of Medicine, Quinnipiac University, Hamden, Connecticut; 5 Robert F. Wagner Graduate School of Public Service, New York University, New York; 6 Population Studies and Training Center, Brown University, Providence, Rhode Island

## Abstract

**Importance:**

Although 23% of births in the US are to non–US-born birthing parents and 6% of births are to undocumented immigrants, many immigrants have limited access to insurance coverage for postpartum care or contraception, which contributes to substantial health disparities. Understanding policy variation in postpartum coverage for immigrants may lay the groundwork for assessing the association of different policy options with maternal and child health.

**Objective:**

To determine if and how each state provides publicly funded insurance coverage during the postpartum period for immigrant birthing parents.

**Design, Setting, and Participants:**

Policy documentation from every US state and Washington, DC, was reviewed, and legal epidemiology policy mapping via a qualitative content analysis of policy documentation was conducted. Data were collected and analyzed on an ongoing basis between June 2021 and February 2024.

**Main Outcomes and Measures:**

For each state, the postpartum funding mechanism for undocumented immigrants and lawful permanent residents were identified, as well as eligibility criteria, services covered, and the duration of eligibility.

**Results:**

A total of 19 states cover some postpartum care with the Children Health Insurance Program (CHIP) from conception to end of pregnancy option, 30 states use Medicaid or CHIP through the CHIP Reauthorization Act Section 214, and 7 states use state-only funds. There was wide variation within each of these mechanisms regarding financial eligibility, services covered, and duration of coverage.

**Conclusions and Relevance:**

The results of this qualitative study suggest that there is substantial variation between states in whether and how immigrants can access publicly funded health insurance during the postpartum period. Further research is needed to examine the association between state policies and postpartum maternal health outcomes.

## Key Points

**Question:**

What are the publicly funded policy mechanisms in each state that cover postpartum care and contraception for immigrants?

**Findings:**

In this qualitative policy analysis, US states covered postpartum care and contraception for immigrants through various mechanisms, including the Children Health Insurance Program (CHIP) from conception to end of pregnancy option, state-only funds, and Medicaid/CHIP through the CHIP Reauthorization Act Section 214, and there was substantial variation within each mechanism.

**Meaning:**

The study results suggest that the wide state variation in funding mechanisms for postpartum care for immigrants may create access challenges in some states but provides opportunities for further policy research.

## Teaser

This qualitative study examines how US states provide publicly funded insurance coverage during the postpartum period for immigrant birthing parents.

## Introduction

In 2021, the US Congress passed the American Rescue Plan Act that, among its many provisions, created a new option for states to extend Medicaid postpartum coverage from 60 days to 12 months using a state plan amendment. This move signaled a broader federal interest in curbing racial disparities in maternal health, and, as of October 2024, 48 states and Washington, DC, have either implemented or plan to implement this expansion. However, the policy does not address access to postpartum care for some groups of immigrants. Although 23% of births in the US are to non–US-born birthing parents, and 6% of births are to undocumented immigrants,^1^ many immigrants have limited access to insurance coverage for postpartum care or contraception.

Federal restrictions on publicly funded insurance for noncitizens who are nonqualified (ie, ineligible for Medicaid due to immigration status) have led many states to develop alternative ways to facilitate postpartum care and contraception access for these populations.^2^ In this article, we examine the policies of all 50 states and Washington, DC, to determine the extent to which each state’s policy facilitates or bars access to affordable postpartum care and contraception for undocumented and documented immigrants who have been in the US for fewer than 5 years. By examining the variation in state policies that provide postpartum coverage for immigrants in the US, we examine the association between state policies and health and insurance coverage outcomes for immigrant birthing parents. In light of the start of the second Trump administration, these findings establish a baseline against which future policy changes can be compared and may also have implications for the creation of and advocacy for ethical and effective health policy in the future.

### Necessity of Postpartum Care and Contraception

Maternal morbidity and mortality are higher in the US than in any other high-income nation,^3,4^ with most pregnancy-related deaths occurring after childbirth.^5^ Postpartum patients are at particularly high risk for several common but treatable health conditions, including postpartum depression and hypertension, and also may choose to initiate postpartum contraception.^6,7,8^ Historically, pregnancy Medicaid coverage has included 60 days of postpartum coverage, which includes at least 1 postpartum visit. While most states do not place limits on the number of postpartum visits, other postpartum benefits vary from state to state, including the length of coverage for postpartum care.^7^ Recent research indicated that 60 days of postpartum care is insufficient to prevent maternal mortality and severe morbidity, as nearly 27% of pregnancy-related deaths occur between 43 days and 1 year postpartum.^5^

During routine postpartum care, which is recommended between 4 and 12 weeks after birth,^8^ patients are tested for postpartum depression and hypertension and receive counseling related to breastfeeding, contraception, and birth spacing.^9^ For patients whose reproductive choices include postpartum contraception, the use of an effective method of contraception during the postpartum period can prevent unintended and short-interval births,^10,11^ both of which are associated with adverse neonatal and maternal health outcomes.^12,13^ People with low incomes, immigrants, and those of racial and ethnic minority groups are disproportionately affected by the maternal health crisis in the US.^9,14,15^ There are also disparities in the use of postpartum contraceptives between non–US-born and US-born people.^16,17^ Previous research has demonstrated that formal exclusions from public insurance coverage contribute to these disparities^18^ and that individual state policies can help to close this gap.^9^

### Immigrant Access to Postpartum Services

Access to publicly funded health care varies widely by immigration status. Lawful permanent residents (LPR), or green card holders, are noncitizens with authorization to live in the US for nonhumanitarian reasons.^19^ To qualify for Medicaid or Children's Health Insurance Program (CHIP) coverage, LPRs must reside in the US for 5 years. States have the option of waiving the 5-year waiting period for children and/or pregnant people with LPR status who would otherwise qualify for coverage under Medicaid or CHIP.^20^ Undocumented immigrants and LPRs present for fewer than 5 years are generally ineligible for most federally funded insurance. Outside of pregnancy, few states offer public insurance coverage to undocumented immigrants, and even in states that offer coverage during pregnancy, this coverage does not always include postpartum care. Under the Emergency Medical Treatment and Labor Act, hospitals that accept Medicare funding (essentially all hospitals in the US) are required to assess and stabilize patients who present to emergency departments experiencing a medical emergency or active labor regardless of their immigration status or ability to pay.^21^ Emergency Medicaid (EM) is available regardless of citizenship or immigration status to cover these costs for patients who meet financial eligibility criteria, but EM does not cover routine health services, including prenatal or postpartum services.^22,23^

As a result, immigrants are much more likely than nonimmigrants to be uninsured. In the low-income population, the percentage of uninsured noncitizen immigrants is 3 times higher than the average among US-born people of reproductive age.^24^ For postpartum noncitizens, these differences are more marked. While 94.2% of citizens and 89.8% of LPRs have insurance coverage during the first 4 months postpartum, only 68.4% of immigrants who are not LPRs do. At 12 months postpartum, these disparities grow to 95.1% of citizens having insurance coverage compared with 86.6% of LPRs and 53.2% of noncitizen/nonpermanent residents.^25^

## Methods

This project used legal mapping methods, which are used in the field of legal epidemiology.^26^ Three members of the study team (A.D., M.W., and K.N.M.) collected policy documentation from each state, including statutes, regulations, clinician manuals, and information sheets created by nonprofit organizations. These documents were analyzed using a qualitative content analysis approach to identify funding mechanisms (eg, CHIP, Medicaid), eligibility criteria (eg, financial, residency status, and duration), and services covered (eg, contraception, postpartum mental health) in each state using the legal mapping software MonQcle, version 3.0 (Temple University).^27^ When discrepancies between state documents arose, the team met to resolve them with further investigation. The findings of our analysis are presented in this article, organized by funding mechanism, and were current through February 1, 2024. Findings are reported according to the Strengthening the Reporting of Observational Studies in Epidemiology (STROBE) reporting guidelines. This project was determined to be exempt from institutional review board review according to federal regulations by the SUNY Upstate Medical University institutional review board.

## Results

**Figure 1.**
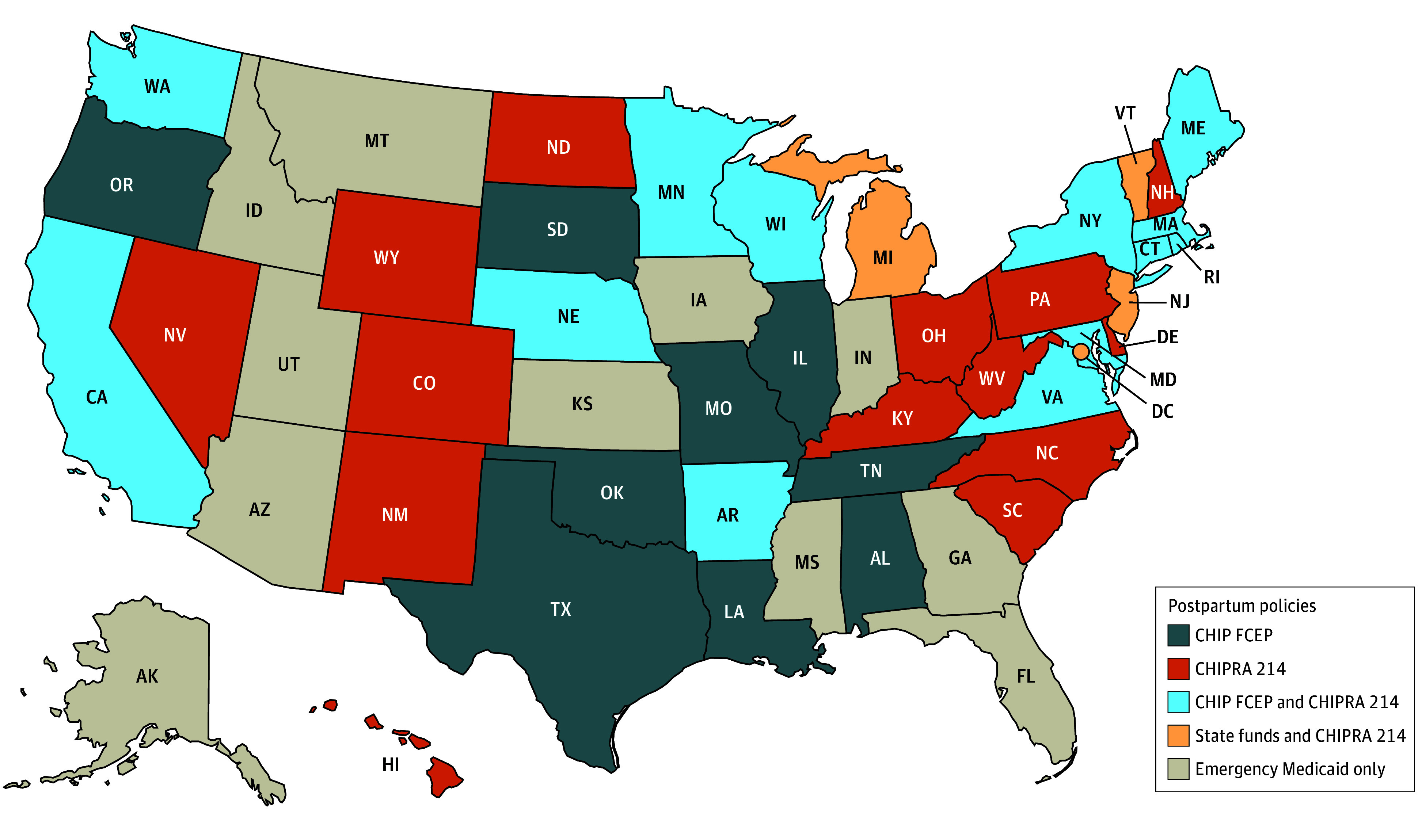
Postpartum Policy Landscape for Children Health Insurance Program (CHIP) From Conception to End of Pregnancy (FCEP), CHIP Reauthorization Act Section 214 (CHIPRA 214; Medicaid or CHIP), and State-Only Funded Programs as of February 1, 2024

**Figure 2.**
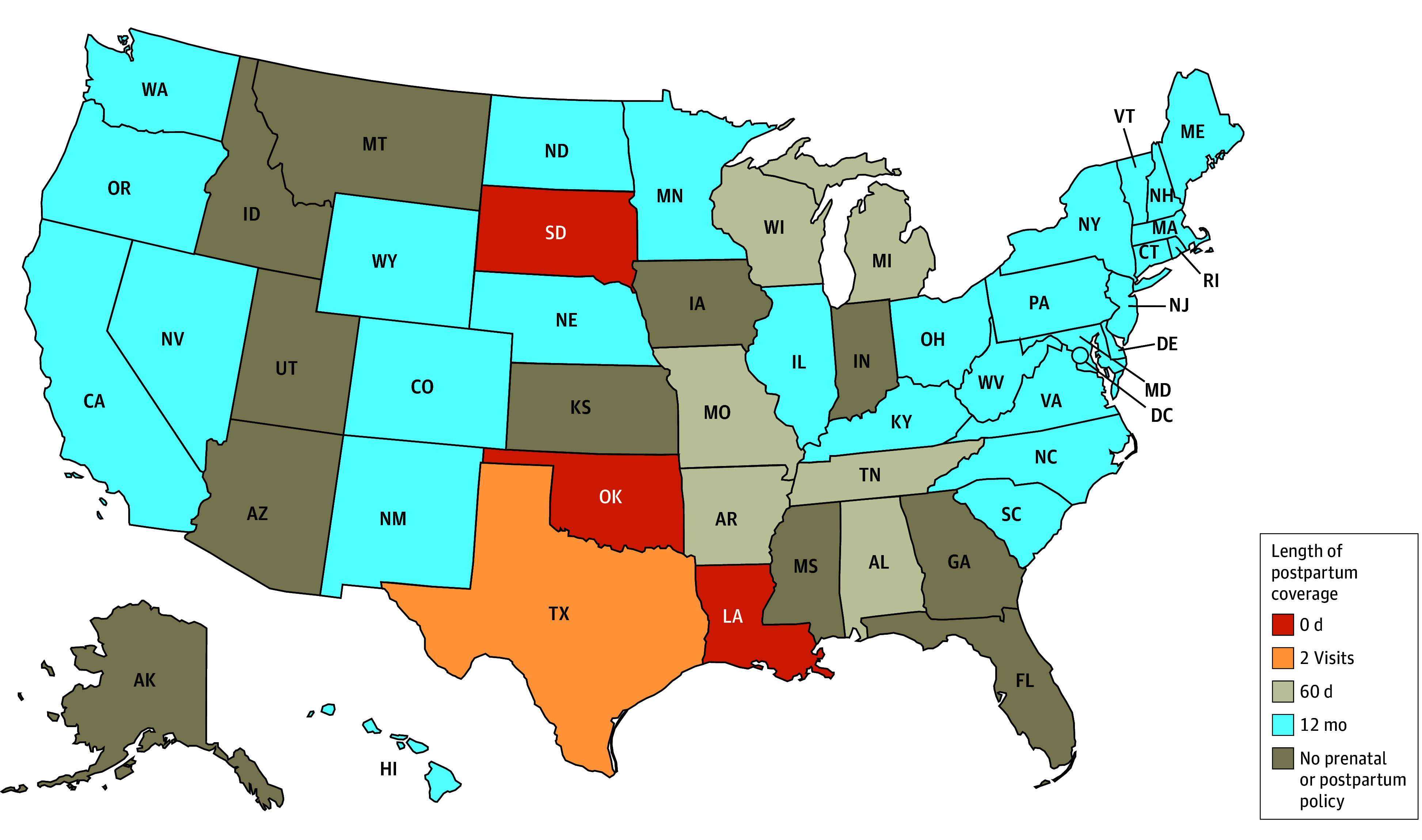
Length of Postpartum Coverage for Lawful Permanent Residents With Fewer Than 5 Years of Residency as of February 1, 2024

**Figure 3.**
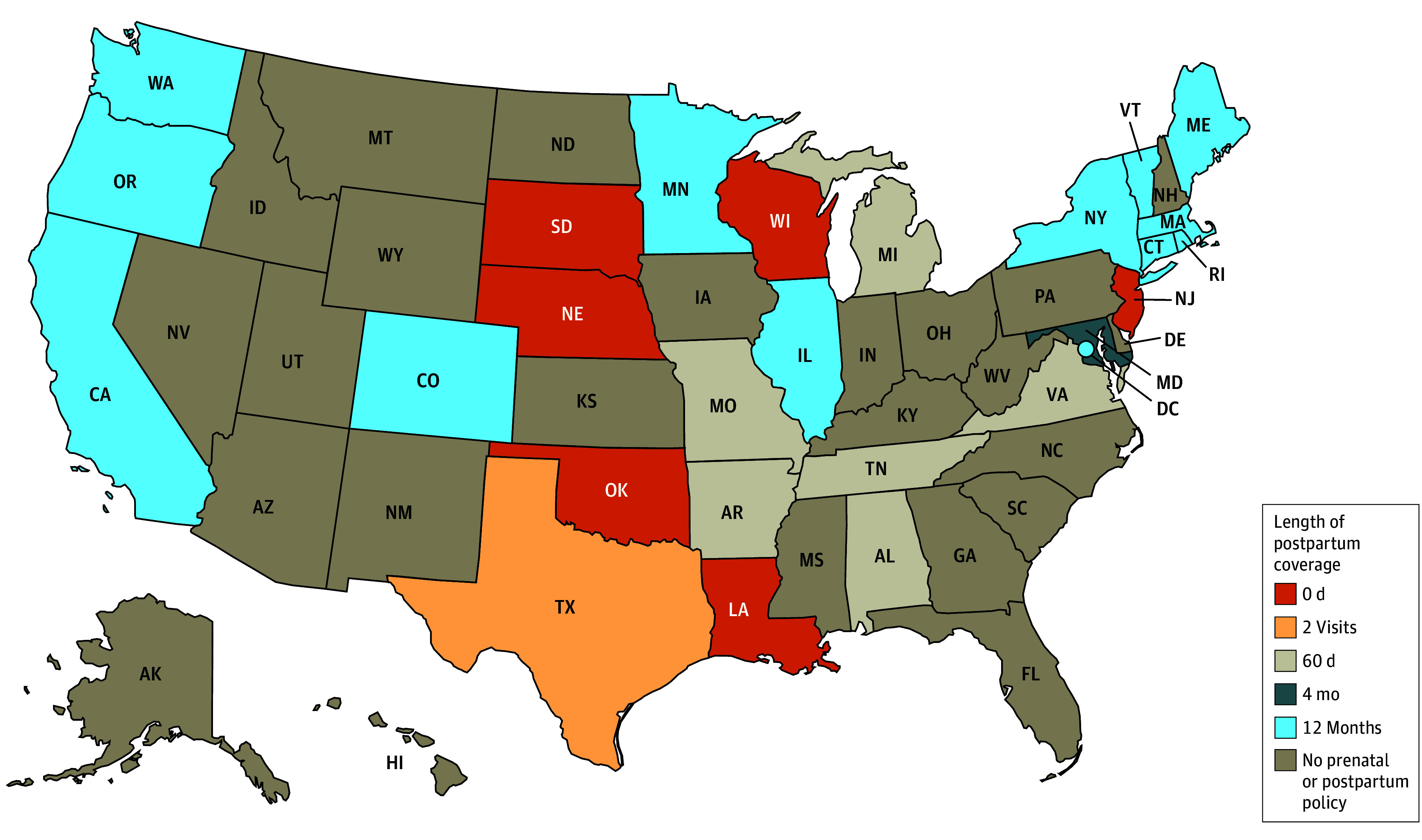
Length of Postpartum Coverage for Undocumented Immigrants as of February 1, 2024

We identified substantial variation in the state policies that provide coverage for postpartum care and contraception to noncitizens. Some states used multiple mechanisms to cover different populations and services, while others had no mechanism to cover postpartum care for these groups. A summary of the various mechanisms is available in the Box that describes the various mechanisms, while Table 1 provides detailed information about the availability of each mechanisms by state (also shown in a US map in Figure 1). Figure 2 and Figure 3 provide information on the length of postpartum coverage by state for LPRs within the 5-year bar and undocumented immigrants, respectively. Finally, Table 2 reports further state program details on the income limits, services covered, and duration of coverage for each group and coverage mechanism.

Box. **Funding Mechanisms for Immigrant Postpartum Care and Contraception**

**CHIP FCEP Option**

States can elect the FCEP option in their Medicaid or CHIP state plans to cover financially eligible pregnant individuals who would otherwise be ineligible for publicly funded insurance due to their immigration status. This option allows states to cover prenatal care and related services that protect the health of the fetus. In some states, this care extends for a period past birth, and some states have adopted CHIP health services initiatives to facilitate this extension.

**State Funds**

State-funded programs vary by state. Some states offer funding for prenatal, postpartum, and/or maternity care, irrespective of immigration status. States may pair state funding of postpartum or other care with one of the other mechanisms to complement their more limited prenatal or postpartum coverage.

**Medicaid/CHIPRA 214**

Medicaid has a federal 5-year waiting period for legal permanent residents to become eligible for benefits. Through CHIPRA, some states chose to expand Medicaid/CHIP coverage to pregnant people within the 5-year bar.

Abbreviations: CHIP, Children Health Insurance Program; CHIPRA 214, CHIP Reauthorization Act Section 214; FCEP, from conception to end of pregnancy.

**Table 1.** State Program Overview of Funding Sources for Various Pregnancy-Related, Postpartum, and Family Planning Services Implemented or Forthcoming as of February 1, 2024

State^a^	CHIP FCEP	Length of FCEP coverage for postpartum care	Medicaid/CHIPRA 214	State-only funds
Alabama	Yes^b^	60 d^c^	NA	NA
Alaska	NA	NA		
Arizona				
Arkansas	Yes	60 d	Yes	
California	Yes	12 mo^c^	Yes	
Colorado	NA^d^	12 mo^c^	Yes	
Connecticut	Yes	12 mo^b^	Yes	
Delaware	NA	NA	Yes	
Florida			NA	
Georgia				
Hawaii			Yes	
Idaho			NA	
Illinois	Yes	12 mo^c^		Yes
Indiana	NA	NA		NA
Iowa				
Kansas				
Kentucky			Yes	
Louisiana	Yes	0 d	NA	
Maine	Yes	12 mo^c^	Yes	
Maryland	Yes	4 mo^c^	Yes	
Massachusetts	Yes	12 mo^c^	Yes	
Michigan	Yes	0 d	NA^d^	60 d Postpartum^d^
Minnesota	Yes	12 mo^c^	Yes	NA
Mississippi	NA	NA	NA	
Missouri	Yes	60 d		
Montana	NA	NA		
Nebraska	Yes	0 d	Yes	
Nevada	NA	NA	Yes	
New Hampshire			Yes	
New Jersey			Yes	Yes^e^
New Mexico			Yes	NA
New York	Yes	12 mo^c^	Yes	Yes
North Carolina	NA	NA	Yes	NA
North Dakota			Yes	
Ohio			Yes	
Oklahoma	Yes	0 d	NA	
Oregon	Yes	12 mo^c^		Yes
Pennsylvania	NA	NA	Yes	NA
Rhode Island	Yes	12 mo^c^	Yes	
South Carolina	NA	NA	Yes	
South Dakota	Yes	0 d	NA	
Tennessee	Yes	60 d		
Texas	Yes	2 Visits		
Utah	NA	NA		
Vermont			Yes	Yes
Virginia	Yes	60 d	Yes	NA
Washington	Yes	12 mo^c^	Yes	Yes
Washington, DC	NA	NA	Yes	Yes
West Virginia			Yes	NA
Wisconsin	Yes	0 d	Yes	
Wyoming	NA	NA	Yes	
Total	23	19	30	7

Abbreviations: CHIP, Children Health Insurance Program; CHIPRA 214, CHIP Reauthorization Act Section 214; FCEP, from conception to end of pregnancy; NA, not applicable.

^a^ All states provide emergency Medicaid.

^b^ Previously, Alabama used a pilot program for the FCEP option using CHIP health services initiative funding to cover prenatal and postpartum care (up to 60 days) in certain counties. This program expanded statewide in April 2024, after the end of the study period.

^c^ State covers extended postpartum period using a CHIP health services initiative or state-only funds.

^d^ The effective dates of policies in Michigan and Colorado began outside the study period; thus, they were excluded from tallies and Table 2.

^e^ The New Jersey Supplemental Prenatal and Contraceptive Program only covers prenatal care and family planning and is not always funded.

**Table 2.** State Program Details

State^a^	Funding source	Eligible groups	Income limit (% FPL)	Services covered	Duration of postpartum coverage
Alabama	CHIP (FCEP)	LPR <5 y, undocumented	317	Prenatal, postpartum care, family planning, L&D	60 d
Arkansas	CHIP (FCEP)	Undocumented	214	Prenatal, postpartum care	60 d
	Medicaid (CHIPRA 214)	LPR <5 y	214	Prenatal, postpartum care, family planning, L&D	60 d
California	CHIP (FCEP)	Undocumented	322	Prenatal, postpartum care, family planning, L&D	12 mo (CHIP HSI)
	Medicaid (CHIPRA 214)	LPR <5 y	213	Prenatal, postpartum care, family planning, L&D	12 mo
Colorado	CHIP (CHIPRA 214)	LPR <5 y	265	Prenatal, postpartum care, family planning, L&D	12 mo
	Medicaid (CHIPRA 214)	LPR <5 y	195	Prenatal, postpartum care, family planning, L&D	12 mo
Connecticut	CHIP (FCEP)	Undocumented	263	Prenatal, postpartum care, family planning, L&D	12 mo (CHIP HSI)
	Medicaid (CHIPRA 214)	LPR <5 y	263	Prenatal, postpartum care, family planning	12 mo
Delaware	Medicaid (CHIPRA 214)	LPR <5 y	217	Prenatal, postpartum care, family planning	12 mo
Hawaii	Medicaid (CHIPRA 214)	LPR<5 y	196	Prenatal, postpartum care, family planning, L&D	12 mo
Illinois	CHIP (FCEP)	LPR <5 y, undocumented	213	Prenatal, postpartum care, L&D	12 mo (CHIP HSI)
Kentucky	Medicaid (CHIPRA 214)	LPR <5 y	195	Prenatal, postpartum care, family planning, L&D	12 mo
	CHIP (CHIPRA 214)	LPR <5 y	218	Prenatal, postpartum care, L&D	12 mo
Louisiana	CHIP (FCEP)	LPR <5 y, undocumented	214	Prenatal, L&D	0 d
Maine	CHIP (FCEP)	Undocumented	213	Prenatal, postpartum care, L&D	12 mo (CHIP HSI)
	Medicaid (CHIPRA 214)	LPR <5 y	214	Prenatal, postpartum care, family planning, L&D	12 mo
Maryland	CHIP (FCEP)	Undocumented	264	Prenatal, postpartum care, L&D	4 mo (CHIP HSI)
	Medicaid (CHIPRA 214)	LPR <5 y	264	Prenatal, postpartum care, family planning, L&D	12 mo
Massachusetts	CHIP (FCEP)	Undocumented	205	Prenatal, postpartum, L&D	12 mo (CHIP HSI)
	Medicaid (CHIPRA 214)	LPR <5 y	205	Prenatal, postpartum care, family planning, L&D	12 mo
Michigan	CHIP (FCEP)	LPR <5 y, undocumented	200	Prenatal, L&D	0 d
	Maternity outpatient medical services (state-only funds)	LPR <5 y, undocumented	200	Prenatal, L&D, postpartum care, family planning	60 d
Minnesota	CHIP (FCEP)	Undocumented	283	Prenatal, postpartum care, family planning, L&D	12 mo (CHIP HSI)
	Medicaid (CHIPRA 214)	LPR <5 y	283	Prenatal, postpartum care, family planning, L&D	12 mo
Missouri	CHIP (FCEP)	Undocumented, LPR <5 y	305	Prenatal, postpartum	60 d
Nebraska	CHIP (FCEP)	Undocumented	202	Prenatal, postpartum, L&D	60 d
	Medicaid (CHIPRA 214)	LPR <5 y	199	Prenatal, postpartum care, family planning, L&D	12 mo
Nevada	Medicaid (CHIPRA 214)	LPR <5 y	190	Prenatal, postpartum care, family planning, L&D	12 mo
New Hampshire	Medicaid (CHIPRA 214)	LPR <5 y	190	Prenatal, postpartum care, family planning, L&D	12 mo
New Jersey	New Jersey Supplemental Prenatal and Contraceptive Program (state-only funds)	Undocumented	205	Prenatal, family planning counseling, birth control	0 d
	CHIP (CHIPRA 214)	LPR <5 y	205	Prenatal, postpartum care	12 mo
	Medicaid (CHIPRA 214)	LPR <5 y	194	Prenatal, postpartum care, family planning	12 mo
New Mexico	Medicaid (CHIPRA 214)	LPR <5 y	255	Prenatal, postpartum care, family planning	12 mo
New York	CHIP (FCEP)	Undocumented	223	Prenatal, postpartum care, family planning, L&D	12 mo (First 60 d under FCEP, remainder with state funds)
	Medicaid (CHIPRA 214)	LPR <5 y	223	Prenatal, postpartum care, family planning, L&D	12 mo
North Carolina	Medicaid (CHIPRA 214)	LPR <5 y	201	Prenatal, postpartum care, family planning, L&D	12 mo
North Dakota	Medicaid (CHIPRA 214)	LPR <5 y	201	Prenatal, postpartum care, family planning, L&D	12 mo
Ohio	Medicaid (CHIPRA 214)	LPR <5 y	205	Prenatal, postpartum care, family planning, L&D	12 mo
Oklahoma	CHIP (FCEP)	LPR <5 y, undocumented	210	Prenatal, L&D	0 d
Oregon	CHIP (FCEP)	LPR <5 y, undocumented	190	Prenatal, postpartum care, family planning, L&D	12 mo (CHIP HSI)
Pennsylvania	Medicaid (CHIPRA 214)	LPR <5 y	220	Prenatal, postpartum care, family planning, L&D	12 mo
Rhode Island	CHIP (FCEP)	Undocumented	258	Prenatal, postpartum care, family planning, L&D	12 mo (CHIP HSI)
	Medicaid (CHIPRA 214)	LPR <5 y	258	Prenatal, postpartum care, family planning, L&D	12 mo
South Carolina	Medicaid (CHIPRA 214)	LPR <5 y	199	Prenatal, postpartum, family planning	12 mo
South Dakota	CHIP (FCEP)	LPR <5 y, undocumented	138	Prenatal, L&D	0 d
Tennessee	CHIP (FCEP)	LPR <5 y, undocumented	255	Prenatal, postpartum care, L&D	60 d
Texas	CHIP (FCEP)	LPR<5 y, undocumented	207	Prenatal, postpartum care	2 visits
Vermont	Immigrant Health Insurance Plan (state-only funds)	Undocumented	213	Prenatal, postpartum, family planning	12 mo
	Medicaid (CHIPRA 214)	LPR <5 y	213	Prenatal, postpartum, family planning	12 mo
Virginia	CHIP (FCEP)	Undocumented	205	Prenatal, postpartum care, L&D	60 d
	Medicaid (CHIPRA 214)	LPR<5 y	143	Prenatal, postpartum care, family planning, L&D	12 mo
	CHIP (CHIPRA 214)	LPR <5 y	205	Prenatal, postpartum care, family planning, L&D	12 mo
Washington	CHIP (FCEP)	LPR <5 y, undocumented	198	Prenatal, postpartum care, L&D	12 mo (CHIP HSI)
	Medicaid (CHIPRA 214)	LPR <5 y	198	Prenatal, postpartum care, family planning, L&D	12 mo
Washington, DC	DC Healthcare Alliance (state-only funds)	Undocumented	215	Prenatal, postpartum care, family planning, L&D	12 mo
	Medicaid (CHIPRA 214)	LPR <5 y	324	Prenatal, postpartum care, family planning	12 mo
West Virginia	Medicaid (CHIPRA 214)	LPR <5 y	185	Prenatal, postpartum, family planning	12 mo
	CHIP (CHIPRA 214)	LPR <5 y	305	Prenatal, postpartum coverage, L&D	12 mo
Wisconsin	CHIP (FCEP)	LPR <5 y, undocumented	306	Prenatal, L&D	0 d
	Medicaid (CHIPRA 214)	LPR <5 y	306	Prenatal, postpartum care, family planning	60 d
Wyoming	Medicaid (CHIPRA 214)	LPR <5 y	159	Prenatal, postpartum care, family planning	12 mo

Abbreviations: CHIP, Children Health Insurance Program; CHIPRA 214, CHIP Reauthorization Act Section 214; FCEP, from conception to end of pregnancy; FPL, federal poverty line; HSI, health services initiative; L&D, labor and delivery; LPR, lawful permanent resident.

^a^ The states described in the table adopted 1 of the 3 mechanisms examined in this article (FCEP, CHIPRA 214, or state-only funding). States missing from this table did not have any of these mechanisms in place, but all states offer emergency Medicaid coverage for financially eligible people who lack insurance, regardless of their immigration status. Emergency Medicaid typically does not cover pregnancy-related services other than L&D.

### Children’s Health Insurance Program

CHIP is a federal and state program that provides low-cost health coverage for children in families that have incomes too high to be eligible for Medicaid but who do not have private insurance. Financial eligibility for CHIP coverage is determined using modified adjusted gross income. Eligibility limits range from 138% of the federal poverty line (FPL) (South Dakota) to 317% of the FPL (Alabama), with a median income threshold of 214% of the FPL.^28^ In some states, pregnant people are also covered by CHIP through a program known as the from conception to end of pregnancy (FCEP) option, which was formerly (and not uncontroversially^29^) known as the CHIP unborn child option.^30^ We found that 23 states cover some services for undocumented pregnant people using the FCEP option, which permits states to consider a fetus a “targeted low-income child” and allows for the provision of pregnancy-related care through CHIP, regardless of immigration status.^31^ Of the 23 states that have the FCEP option, 5 states (Louisiana, Nebraska, Oklahoma, South Dakota, and Wisconsin) use CHIP FCEP to cover prenatal services only or prenatal services and labor and delivery for undocumented people. The other 18 states use either a bundled payment model, state-only funds, or a CHIP health services initiative (HSI) to cover postpartum services after birth, such as a postpartum physician appointment or outpatient mental health services.

Among FCEP states that cover postpartum services, there is wide variability in the duration of coverage and type of postpartum services included. Eleven states cover postpartum services through 12 months, while 7 states include coverage for a shorter period (2 visits: Texas; 60 days: Arkansas, Michigan, Missouri, Tennessee, and Virginia; 4 months: Maryland).

### State-Only Funding

Some states use state-only funds (ie, money from the state budget that is not matched by the federal government) to cover select services due to the restrictions around how federal funds can be used for nonqualified immigrants. While 6 states and Washington, DC, cover some groups of federally nonqualified immigrants outside of pregnancy (California, Colorado, Washington, DC, Illinois, New York, Oregon, and Washington), many of these programs are constrained by funding limitations and regularly close enrollment^32^ or only cover people older than 65 years. There are 2 states (Vermont and New Jersey) that cover pregnancy-related care with state-only funds, although they diverge substantially on what benefits are covered. Michigan uses state-only funds to cover 60 days of postpartum care for the group whose pregnancy is covered by FCEP.

### Medicaid/CHIP Reauthorization Act Section 214

While CHIP FCEP and state-funded programs can use funds to cover undocumented immigrants, states are prohibited from using Medicaid funds for this population. However, states may elect to use Medicaid or CHIP funds to cover LPRs within the 5-year bar. The policy granting states this option was created by section 214 of the Children’s Health Insurance Program Reauthorization Act of 2009 (CHIPRA 214).^33^

Through our analysis, we found that 29 states and Washington, DC, use the CHIPRA 214 option to cover postpartum care for LPRs through either their state Medicaid or the CHIP program. Because Medicaid and CHIP financial eligibility and services vary substantially by state, eligibility for and benefits covered under CHIPRA 214 are similarly varied, nonuniform, and mirror state variation in traditional Medicaid and CHIP programs. People eligible under CHIPRA 214 receive the same (varied) full-scope Medicaid/CHIP benefits as other pregnant or postpartum beneficiaries in their state, and states that adopt the 12-month postpartum Medicaid extension for traditional Medicaid populations also must do so for LPRs covered through CHIPRA 214.^34^ Of the 30 states that extend coverage to lawfully residing pregnant people under CHIPRA 214, all but 1 (Arkansas) have adopted the 12-month postpartum extension.

## Discussion

In this qualitative study, the state policies on postpartum care for undocumented immigrants and LPRs with fewer than 5 years of residence revealed commendable initiatives but persistent disparities. These differences in coverage may have substantial implications for maternal and child health, as well as uptake of contraception after and between pregnancies. In states that offer no public coverage beyond EM, nonqualified immigrants may have little access to outpatient health care to monitor and treat common postpartum health conditions, such as hypertension, depression, urinary and breast infections, and pain.^25^ Delayed care could be associated with increased severity of these conditions, followed by worse outcomes and hospital readmission. Additionally, states that have implemented a CHIP HSI to extend a year of postpartum coverage to people enrolled in the FCEP program during pregnancy are able to cover the period during which maternal mortality most frequently occurs; states with only FCEP coverage and 60 days or fewer of postpartum coverage are not. This policy variation has created confusion among pregnant immigrants and presents practical challenges to summarizing the national state of pregnancy coverage for immigrants.^2^ The health effects of this variation will be an important area for future research.^18,35^

Ensuring equitable access to reproductive care for undocumented immigrants is crucial in addressing disparities and promoting reproductive justice, which requires that individuals have the ability to choose to have or not have children and parent their children in safe and healthy environments.^36^ In addition to reducing insurance policy barriers to care, states should also ensure that pregnant and parenting immigrants can access comprehensive, language-concordant information about their postpartum care and contraceptive options. We found very little publicly available information documenting the types of postpartum contraception, including sterilization options, that may be available to immigrants in each state. A lack of information about covered contraceptive methods under the postpartum policies described in this article creates barriers for undocumented immigrants to obtain accessible and accurate information about their contraceptive options.

### Limitations

This study had several limitations. First, the reliance on state documentation and available materials introduced potential gaps and inconsistencies across states, and these materials were often challenging to locate and verify. Additionally, this study presented a static analysis that may not capture the dynamic changes in policies; policy information presented in this article was current as of February 1, 2024, but the landscape has continued to shift after the study period. For instance, Michigan adopted the CHIP 214 option effective later in 2024, and Colorado is extending coverage to pregnant and postpartum people regardless of immigration status starting in 2025; therefore, both policies were excluded from this report. However, despite this limitation, the policy information presented potentially represents a first step toward future research on the health and economic effects of these policies, which will require understanding the policy in place at multiple points. An additional limitation was that, although this analysis was limited to state options, there are also many county-level and city-level programs that we did not consider. Moreover, we did not examine variation in state receipt of federal funding (eg, Title V and Title X funding) for safety net health care services, which are an important source of reproductive health care for uninsured individuals.

## Conclusions

This qualitative study describes policy variations between states, and examining the association between these policies and longer-term health outcomes for immigrant parents may yield insights into the downstream health and economic effects associated with increasing access to publicly funded postpartum care. State variation makes it possible to study the associations of different policies with health outcomes for immigrant parents and children. The differential associations of these various policies with birth, postpartum, and lifetime health outcomes should be studied to determine the best policy approach. Evidence-based policy requires rigorous data analysis and study, and this article represents a potential first step toward understanding state postpartum policies, which will be necessary for state-by-state analysis.

The structure of the US health care system is by its nature fragmented and piecemeal, with each state responsible for the design and implementation of its own safety net and state Medicaid program. This patchwork of inconsistent and variable policies is nowhere more obvious than in the provision of, or exclusion from, health care for pregnant and postpartum noncitizens. If the US is to grapple with its maternal health in a meaningful and sustainable way, this process must include a rigorous evaluation of the state policies affecting postpartum care and contraception among immigrant parents.

## Notes

Supplement. Data sharing statement
