# Supplementary material for: Public Coverage of Postpartum Services for Immigrants
Source: JAMA Health Forum. 2025 Apr 25;6(4):e250702. doi: 10.1001/jamahealthforum.2025.0702 (PMC12032572; doi:10.1001/jamahealthforum.2025.0702)
Supplement: Supplement. — Data sharing statement [file jamahealthforum-e250702-s001.pdf]

# Data Sharing Statement

Fabi. Public Coverage of Postpartum Services for Immigrants. *JAMA Health Forum*. Published April 25, 2025. doi:10.1001/jamahealthforum.2025.0702

**Data**
**Data available:** Yes
**Data types:** Data (not involving human participants)
**How to access data:** fabir@upstate.edu
**When available:** With publication

**Supporting Documents**
**Document types:** None

**Additional Information**
**Who can access the data:** anyone requesting the data
**Types of analyses:** any purpose
**Mechanisms of data availability:** with investigator support
